# Comparison of postoperative ciliary body changes associated with the use of 23-gauge and 20-gauge system for pars plana vitrectomy

**DOI:** 10.1186/s12886-018-0925-9

**Published:** 2018-10-11

**Authors:** Meng-su Tang, Shu-qi Zhang, Li-wei Ma

**Affiliations:** 1 Department of Ophthalmology, the Fourth Affiliated Hospital of China Medical University, No. 11 Xinhua Road, Heping District, Shenyang, 110004 Liaoning Province China; 2 Department of Ophthalmology, the 463 Hospital of the Chinese People’s Liberation Army, Shenyang, 110021 Liaoning Province China

**Keywords:** Pars plana vitrectomy, Ciliary body, Idiopathic epiretinal membrane, 23-gauge system

## Abstract

**Background:**

To compare the ciliary body changes associated with the use of 23-gauge (23G) and 20-gauge (20G) systems for pars plana vitrectomy.

**Methods:**

A total of 60 patients (60 eyes) with idiopathic epiretinal membrane who were scheduled for surgical treatment were selected and randomly assigned to 20G group or 23G group. Time required for incision making, vitrectomy, and incision closure was compared between the two groups. Changes in ciliary body were evaluated by ultrasound microscopy (UBM). Anterior chamber inflammation was assessed with laser flare meter instrument.

**Results:**

Incision-making time (4.5 ± 0.9 min) and incision-closure time (2.8 ± 0.7 min) in the 23G group were significantly shorter than those in the 20G group (10.1 ± 1.5 min and 11.3 ± 2.2 min, respectively). No significant intergroup difference was observed with respect to time required for vitrectomy (21.6 ± 3.3 min and 20.7 ± 3.2 min, respectively). Ciliary body thickness in the 23G group recovered back to preoperative levels after 4 weeks, as against 8 weeks in the 20G group. Postoperative ciliary body thickness in the 20G group was significantly higher than that in the 23G group (*p* < 0.05). The aqueous protein concentration in 23G group recovered back to preoperative levels after 2 weeks, as against 4 weeks in the 20G group. Postoperative aqueous protein concentration in the 20G group was significantly higher than that in the 23G group (*p* < 0.05).

**Conclusions:**

The use of 23G system was associated with significantly milder injury to the ciliary body as compared to that associated with the use of 20G system.

**Trial registration:**

The study was retrospectively registered on Chinese Clinical Trial Registry. The clinical study registration number was ChiCTR-INR-17011082. Date of registration: 2017-04-07.

## Background

Technological advancements in vitreoretinal surgery have made it possible to treat certain diseases which were hitherto considered untreatable. Pioneered by Machemer in the early 1970s, the pars plana vitrectomy technique has evolved into an increasingly advanced minimally-invasive treatment modality. Introduced for the first time by Claus Eckardt in 2005, the 23-gauge (23G) transconjunctival vitrectomy technique is commonly used by vitreoretinal surgeons in daily practice. The advantages of use of 23G system include a smaller incision, milder inflammatory response, and more rapid recovery [1–5]. However, use of 23G system may lead to low intraocular pressure, choroidal detachment, and incisional vitreous incarceration in the short term after the procedure [6–8].

Pars plana vitrectomy is a safe procedure used to manage vitreoretinal disease. However, it could also alter the anterior segment morphology and increase the risk of early postoperative complications such as transient decrease in depth of anterior chamber, angle narrowing, persistent hypotony, and supraciliary effusion [9–11]. The underlying retinal microangiopathy increases the risk of postoperative inflammation, uveal congestion, and changes of the ciliary body in patients with vitreoretinal disease; these changes may be severe in some cases. However, clinical evidence pertaining to post-vitrectomy changes of the ciliary body in such patients is not well documented.

Ultrasound biomicroscopy has been used to monitor postoperative incision healing after use of 20G and 25G vitrectomy systems, [12–15] by serial measurements of the thickness of the ciliary body. In addition, laser flare meter provides a non-invasive means for quantitative monitoring of blood-aqueous barrier function, through laser reflection of the anterior pupil area to indicate the aqueous protein concentration; the technique allows for evaluation of ciliary body wound healing from another aspect [16–18]. In this study, changes of ciliary body thickness and aqueous protein concentration before and after operation were studied to explore the impact on 23G and 20G vitrectomy.

## Methods

### Objects

A total of 60 patients (60 eyes) with idiopathic epiretinal membrane, who underwent surgical treatment at our hospital during January 2016 and February 2017 were selected. The patients included 22 men and 38 women (age range, 39 to 60 years). The subjects were randomly assigned to 23G group or 20G group using a random number table. No statistical difference was observed on age and gender distribution between two groups. Patients in the 23G group received 23G minimally invasive vitrectomy and those in the 20G group received 20G traditional standard three-channel vitrectomy. Written informed consent was obtained from all patients prior to their enrolment. The study was approved by the institutional ethics committee. The clinical study registration number was ChiCTR-INR-17011082.

Inclusion criteria: (1) idiopathic epiretinal membrane diagnosed based on optical coherence tomography and fundus fluorescein angiography; (2) best corrected visual acuity ≤0.3; (3) diopter between ±3.0 D.

Exclusion criteria: (1) history of eye surgery; (2) glaucoma, familial glaucoma history, or intraocular hypertension; (3) uveitis; (4) severe lenticular opacity requiring cataract extraction; (5) need for intraocular tamponade, such as silicone oil or gas; (6) patients with diabetes, hypertension, and autoimmune conditions, such as rheumatoid arthritis, systemic lupus erythematosus, and multiple sclerosis.

### Operation method

All patients were operated under general or local anesthesia by the same surgeon (L.M.). Vitrectomy machine system (ACCURUS, ALCON) was used for 23-gauge vitrectomy and 20-gauge vitrectomy. In the 23G group, the conjunctiva was pushed laterally using a pressure plate. Subsequently, two-step tunnel-like transconjuctival incisions were made using a sharp 23G blade at a 20–30° angle parallel to the limbus facilitating insertion of the trocars. After vitrectomy, the trocars were removed and the sclerotomies were covered by the conjunctiva.

In the 20G group, the conjunctiva was opened in a nasal triangle and a temporal quadrangle 1 mm from the limbus followed by scleral incisions 3.5–4 mm behind the limbus without electrocoagulation. After vitrectomy, the sclerotomies and conjunctiva were closed with vicryl 8.0 sutures. In both groups, a thorough vitrectomy was performed with the goal of removing vitreous. Time required for incision-making, vitrectomy, and - incision-closure was recorded for all patients.

All patients were administered topical levofloxacin 6 times per day and atropine ophthalmic gel 2 times per day from 3 days before the operation. After the operation, the subjects received topical tobramycin dexamethasone 4 times per day and atropine ophthalmic gel 2 times per day. The tobramycin dexamethasone was tapered off over 4 weeks and changed to pranoprofen 4 times per day for another 4 weeks. Atropine ophthalmic gel was stopped after 2 weeks.

### Postoperative observation

The evaluation of ciliary body by ultrasound microscopy (UBM) (ODM-3000, Tianjin Meda Medical Technology Co., Ltd.) was performed by the same technician. The ciliary body thickness at three incisions was measured to obtain the mean value. Each measurement was repeated three times to calculate the mean value. Ethylene oxide was used to sterilize the eye cup and absolute alcohol was adopted to wipe the UBM probe. Anterior chamber inflammation was detected by laser flare meter(FM600, Kowa) to measure the aqueous protein concentration. Each measurement was repeated three times to calculate the mean value. All subjects were assessed with UBM and laser flare meter preoperatively, on postoperative day 1, week 1, week 2, and week 4. UBM examination was performed again at the postoperative week 8.

### Statistical analysis

All data analyses were performed with SPSS 19.0 software(IBM Corporation, NY). The ciliary body thickness and aqueous protein concentration before and after the operation were compared by two-way ANOVA. Ciliary body thickness, aqueous protein concentration, and operation time of the two groups were compared by *t* test. *p* < 0.05 was considered statistically significant.

## Results

### General information

The baseline demographic data of the two groups were listed in Table 1. The lens was not exceed C1N1P0 according to LOCSII classification.

**Table 1:** Patient demographic data

	23G	20G
Cases(eye)	30	30
Mean age	52	51
Age range	39—60	41—60
Male	10	12
Female	20	18
preoperative ciliary body thickness (mm)	0.25 ± 0.02	0.25 ± 0.02
preoperative aqueous protein concentration (pc/ms)	6.7 ± 1.6	6.9 ± 1.4

### Complications

Eight patients in the 23G group and six patients in the 20G group developed punctate hemorrhage in the macular area. The bleeding was self-absorbed without laser or electrocoagulation intervention. The lens was retained in all patients, and no intraocular tamponade was used at the end of surgery. Six patients in the 23G group experienced mild choroidal detachment on the first day after operation, which recovered in one week. However, none of the patients in the 20G group experienced choroidal detachment. Fifteen patients in the 23G group and two patients in the 20G group had low intraocular pressure on the first postoperative day and recovered in one week. Vitreous incarceration was observed in 3(10%, 23G) and 0(0%, 20G) eyes on the first postoperative day. None of the patients developed postoperative endophthalmitis.

### Time for surgery

The 23G group exhibited significantly shorter time for incision-making (4.5 ± 0.9 min) and incision-closure (2.8 ± 0.7 min) as compared to that in the 20G group (10.1 ± 1.5 min and 11.3 ± 2.2 min), respectively (*t* = 17.771, *p* < 0.05; *t* = 19.868, *p* < 0.05). No statistically significant intergroup difference was observed with respect to time for vitrectomy (21.6 ± 3.3 min vs. 20.7 ± 3.2 min, *t* = 1.038, *p* > 0.05).

### Measurement of ciliary body thickness

**Fig. 1:**
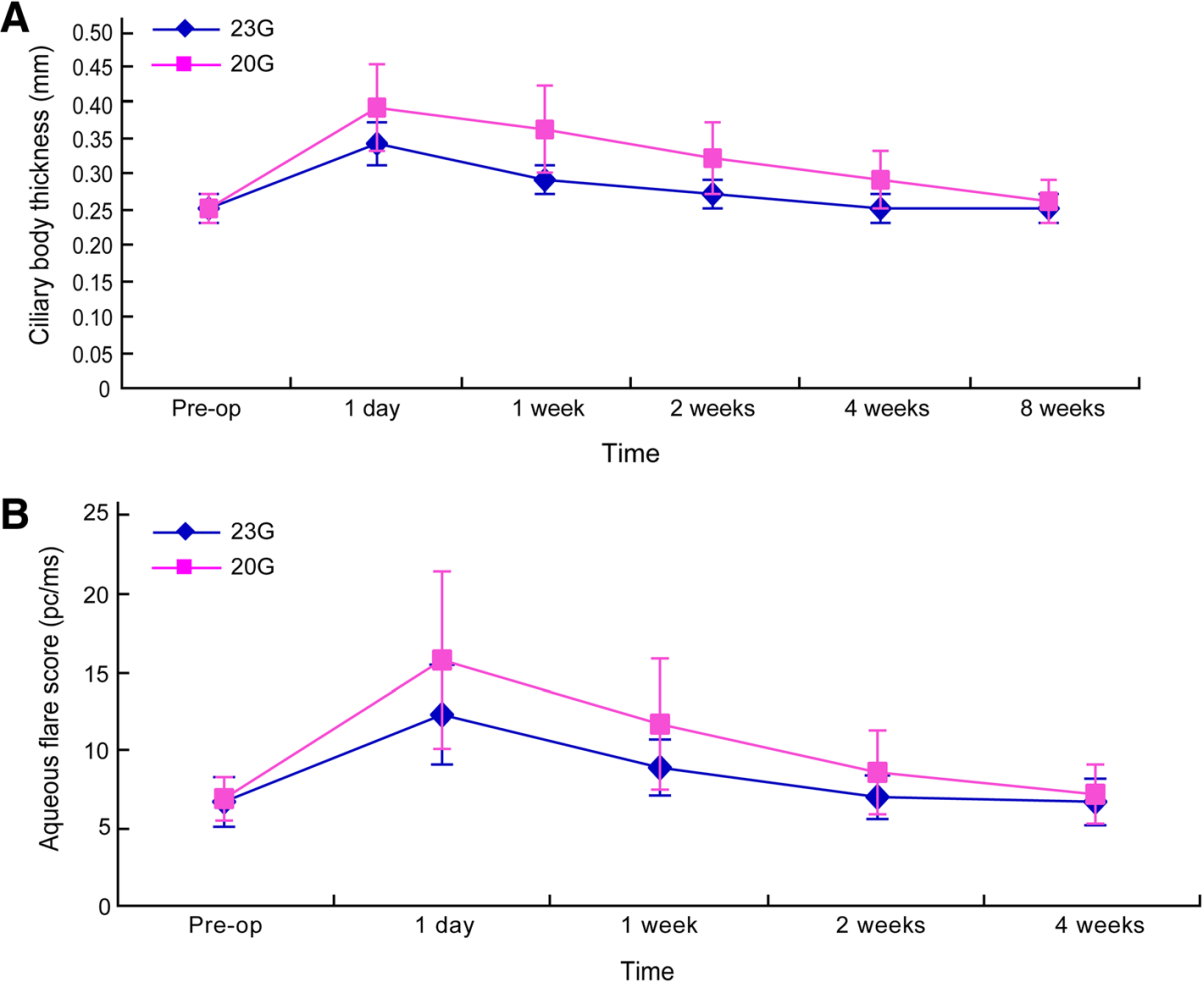
Postoperative changes of ciliary body thickness and aqueous flare score. **a**, ciliary body thickness. **b**, aqueous flare score

**Fig. 2:**
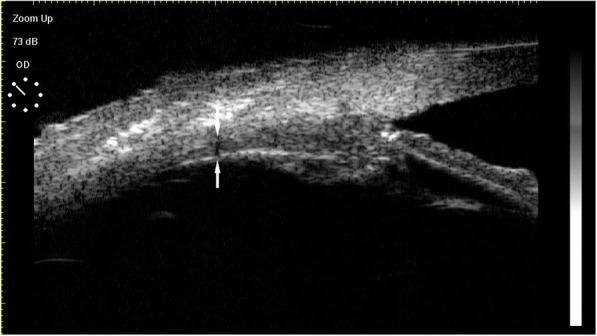
Ciliary body thickness in 20 G group at 4 weeks after surgery detected by UBM (arrow)

As shown in Fig. 1a, no significant intergroup difference was observed with respect to preoperative ciliary body thickness (*t* = 0.064, *p* > 0.05). A significant increase in ciliary body thickness was observed postoperatively in both groups (*F* = 263.83, *p* < 0.05). Ciliary body thickness in the 23G group recovered to preoperative levels after 4 weeks as against 8 weeks in the 20G group. Postoperative ciliary body thickness in the 20G group was significantly higher than that in the 23G group (F = 18.913, *p* < 0.05). The measurement method of ciliary body thickness was exhibited in Fig. 2.

### Measurement of aqueous protein concentration

As shown in Fig. 1b, no significant intergroup difference was observed with respect to preoperative aqueous protein concentration (*t* = 0.592, *p* > 0.05). A significant increase in aqueous protein concentration was observed postoperatively in both groups (F = 117.246, *p* < 0.05). Aqueous protein concentration in the 23G group recovered to preoperative levels in 2 weeks as against 4 weeks in the 20G group. Aqueous protein concentration in the 20G group was significantly higher than that in the 23G group (F = 7.775, *p* < 0.05).

## Discussion

Minimal-invasive procedures are the tendency of modern ocular surgery. According to many previous studies, sutureless vitrectomy has its benefits and risks. The sutureless vitrectomy system shortens the operation time to some extent, thus providing quicker recovery time. However, low intraocular pressure and ciliary body detachment may appear in the early stage of postoperative period [19–21].

In our study, small-incision vitrectomy(23G) was associated with shorter operation time and faster postoperative recovery. This mirrors the results of previous studies [19–21]. 23G system with the wider cutter opening and much closer to the cutter head can not only increase the cutting rate, but also reduce the unnecessary disturbances to the retina which makes up its lower efficiency due to the thinner pipe. This is proven by the result of our study that the vitrectomy time was no significant difference in 20G and 23G groups. Due to the shorter incision-making and incision-closure time, the total operation time was much shorter in 23G group..

Previous studies mainly focused on ciliary body detachment and vitreous incarceration. However, it is less investigated about the impact of the two systems on the thickness of ciliary body. In this study, we focused on the quantative change of ciliary body at the incision site. In addition, all the patients retained the lens, which helped avoid thermal damage caused by phacoemulsification. Furthermore, no silicone oil or gas tamponade were used in these cases, which avoids the impact of intraocular tamponade on the ciliary body. The ciliary body changes were mainly caused by the two different vitrectomy systems. The results of this study indicated postoperative increase in thickness of the ciliary body in both the groups; however, the amplitude of increase in the 23G group was significantly lower than that in the 20G group. Additionally, the ciliary body thickness recovered to the preoperative level after two weeks in 23G group, as compared to 4 weeks in the 20G group, which suggests that 23G vitrectomy system caused lesser damage to the ciliary body. This is attributable to several factors. Firstly, compared with the traditional 20G system, 23G vitrectomy system enters the eye through the trocar fixed on pars plana of the ciliary body, without direct contact with the sclera and the ciliary body. The trocar helped avoid the direct contact of surgical instruments with the ciliary body. The lesser friction of the surgical instruments minimized the mechanical damage to the ciliary body. Secondly, the presence of a part of the trocar in the eye effectively reduces intraoperative vitreous spillover and the traction effect of the ciliary body in the vicinity of the incision site. In the 20G group, 4 patients, who were found that part of the pigment epithelium of ciliary body was brought out together with the spilled vitreous, experienced a particularly high degree of postoperative increase in ciliary body thickness. Thirdly, a conjunctival oblique incision (two-step method) was applied in 23G group, which was subject to automatic closure and required no suture. On the contrary, incisions in the 20G group were closed by absorbent suture. Granulomatous inflammation during degradation of the suture may affect the repair of the ciliary body, and prolong the time required for recovery of ciliary body. Though previous studies did not involve direct observation of ciliary body damage after pars plana vitrectomy, several studies indicate that unsutured vitrectomy is associated with lesser postoperative inflammation and shorter recovery time, which is consistent with our results [22, 23].

The blood-aquoeus barrier function is disturbed after trauma or surgery. The operation-induced blood-aquoeus barrier dysfunction may be related to mechanical damage and thermal injury.. None of the patients in this study received retinal photocoagulation treatment or cataract phacoemulsification, which helped exclude the impact of thermal injury. Blood-aquoeus barrier dysfunction may be mainly caused by mechanical damage in the anterior uvea. We employed laser flare meter to measure aqueous protein concentration in order to assess the degree of damage to the blood-aquoeus barrier function. Smaller incisions for cataract surgery have been shown to attenuate damage to the blood-aquoeus barrier function as compared to larger incisions [24, 25]. In this study, postoperative aqueous protein concentrations were increased to varying degrees in both the groups, which indicates that all patients suffered from blood-aquoeus barrier function damage because of mechanical injury. However, the increasement of aqueous protein concentration in the 23G group was significantly smaller than that in the 20G group, which suggests that mechanical injury to the ciliary body in the 23G group was lesser than that in the 20G group. In addition, the time required for aqueous protein concentration recovery was 2 weeks in the 23G group and 4 weeks in the 20G group, which further demonstrates that 23G vitrectomy system was relatively less invasive.

Besides the benefits of sutureless vitrectomy discussed above, there still some risks of it are worth of attention. The incidence of hypotony and choroidal detachment in the 23G group was significantly higher than that in the 20G group, which was associated with intraoperative fluid filler and postoperative incision leakage [26, 27]. According to our results, the higher incidence of hypotony and choroidal detachment in 23G group mostly happened in the early postoperative stage, and this transient phenomenon can be restored in about 1 week. While the thickness of ciliary body recovered in 4 weeks in 23G group compared with 8 weeks in 20G group, the transient postoperative hypotony and choroidal detachment did not affect the recovery of ciliary body’s morphology and function. Theoretically, sutureless surgical procedures are associated with a higher risk of endophthalmitis. However, no case of endophthalmitis occurred in our study, although the number of patients is too small to draw any definitive conclusions in this respect.

## Conclusions

In summary, compared with the 20G vitrectomy system, the 23G vitrectomy system apparently reduced the total operation time owing to faster incision-making and closure. The use of the 23G system was associated with significantly less damage to the ciliary body as compared to the use of 20G system.

## Abbreviation

UBM: Ultrasound microscopy
